# A sustainable HPLC method coupled with diode array detection for versatile quantification of telmisartan, chlorthalidone and amlodipine in a fixed-dose antihypertensive formulation and dissolution studies

**DOI:** 10.1186/s13065-024-01276-2

**Published:** 2024-09-12

**Authors:** Mona A. Kamel, Christine K. Nessim, Adel M. Michael, Samah S. Abbas, Hoda M. Marzouk

**Affiliations:** 1 Pharmaceutical Chemistry Department, Faculty of Pharmacy, Ahram Canadian University, 6th of October City, Cairo 12566 Egypt; 2 Pharmaceutical Analytical Chemistry Department, Faculty of Pharmacy, Cairo University, Kasr El-Aini Street, Cairo, 11562 Egypt

**Keywords:** Amlodipine, Chlorthalidone, Greenness evaluation tools, HPLC–DAD, Telmisartan, Whiteness & Blueness Assessments

## Abstract

Cardiovascular diseases, especially hypertension, stand as prominent contributors to global mortality. Hypertension, often referred to as a silent killer syndrome, necessitates the use of multiple medications for effective control and management. A new environmentally friendly HPLC–DAD method is introduced in this study for the concurrent analysis of telmisartan (TEL), chlorthalidone (CHT) and amlodipine besylate (AML), in both pure forms and combined pharmaceutical dosage form. An isocratic elution mode was employed to achieve chromatographic separation, utilizing an Inertsil C_18_ column (250 × 4.6 mm, 5.0 µm) and a mobile phase mixture of acetonitrile and phosphate buffer (pH 3.0 ± 0.1) with ratio of 35:65, v/v. The separation was achieved within 10 min at a flow rate of 1.0 mL/min. The proposed method's validation was carried out following the guidelines outlined by the International Council for Harmonisation (ICH). The achieved linearity range was 1.0–140.0 μg/mL for TEL and 1.0–100.0 μg/mL for CHT and AML with quantification limits of 0.061, 0.177, and 0.313 μg/mL for TEL, CHT, and AML, respectively. The fixed combination tablet dosage form demonstrated acceptable release profile, as indicated by the in-vitro dissolution studies. The studied dissolution media were phosphate buffer pH 7.5, 0.01 N HCl, and water, utilizing a USP type II apparatus at 37 ± 0.5 °C with a stirring rate of 75 rpm. The proposed method was applied successfully for the quality assessment of Telma-ACT® Tablets with good precision and accuracy. Various tools were used for evaluating the level of greenness, including Green Analytical Procedure Index (GAPI), Analytical Greenness Metric for Sample Preparation (AGREEprep), Analytical Eco-Scale (AES), and Analytical Method Greenness Score (AMGS). These tools had confirmed the eco-friendliness of the proposed method. Additionally, the newly introduced White Analytical Chemistry (WAC), and the Blue Applicability Grade Index (BAGI) have been specifically developed to evaluate the sustainability and the applicability of the method.

**Supplementary Information:**

The online version contains supplementary material available at 10.1186/s13065-024-01276-2.

## Introduction

Cardiovascular disorders, including hypertension, are responsible for a significant number of fatalities globally [1]. The effective management of this condition necessitates the use of multiple drug therapies. About one-fourth of all the people in the world have health problems caused by high blood pressure, and it is expected that more people will join this group in the future. Hypertension is one of the life-threatening conditions that lead to death among both elderly and young population [1]. Therefore, different pharmaceutical dosage forms are established and developed for management of patients with hypertension. The recent clinical studies showed that the strategy of using three-in-one pill has advantages over the usual care. The three-in-one pill is more effective, lower in cost and more convenient for patients [2]. A fixed-dose combination of telmisartan (TEL), chlorthalidone (CHT), and amlodipine besylate (AML) has recently been developed as a three-in-one pill for controlling hypertension [3].

**Fig. 1:**
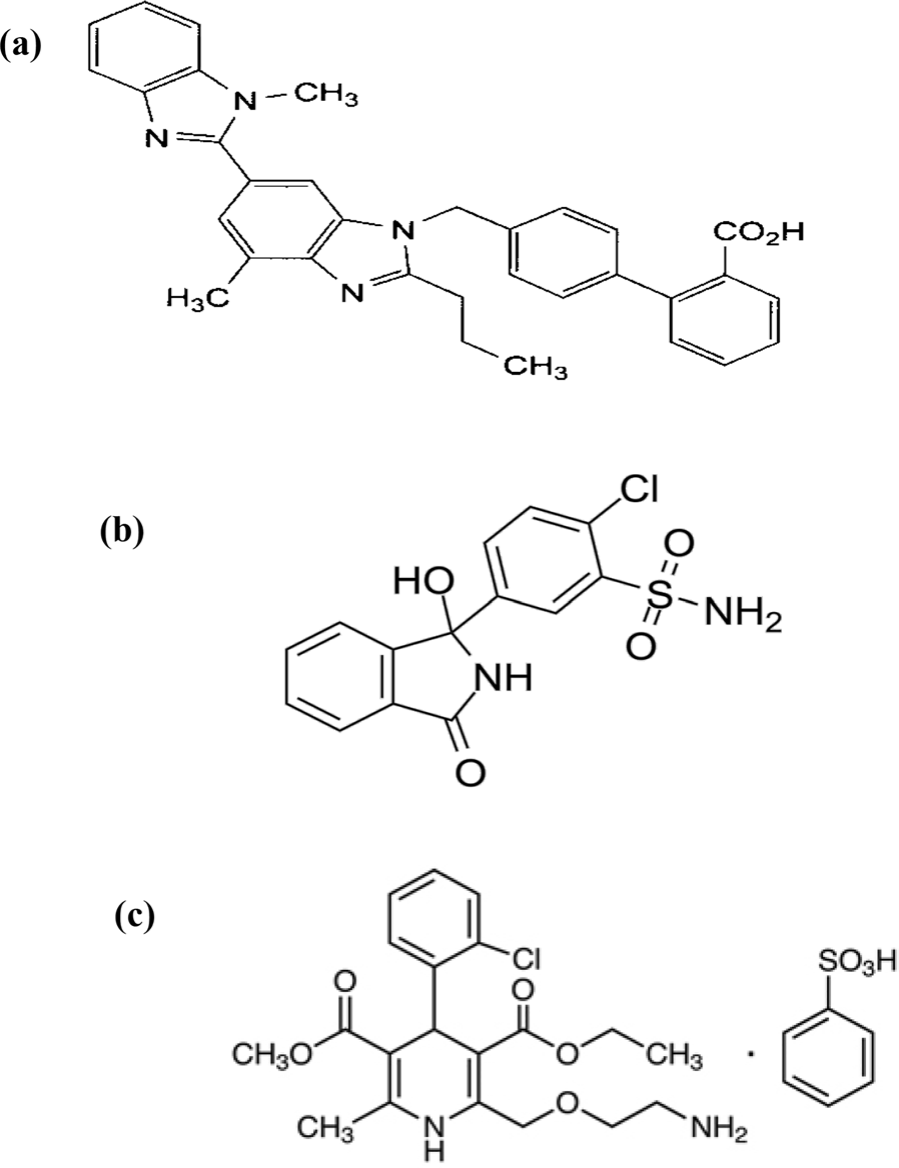
Chemical structures of **a** Telmisartan (TEL), **b** Chlorthalidone (CHT) and **c** Amlodipine besylate (AML)

The studied drugs are official in the British Pharmacopoeia (BP) [4], and the United States Pharmacopeia (USP) [5]. TEL (Fig. 1a) is 4′-[[4-Methyl-6-(1-methyl-1H-benzimidazol-2-yl)-2-propyl-1H-benzimidazol-1- yl] methyl] biphenyl-2-carboxylic acid [4]. TEL belongs to a class of angiotensin receptor blockers that selectively and reversibly bind to the angiotensin II -receptor type 1, blocking it and thereby decreasing systemic vascular resistance [6]. AT1 receptor blockade dilates blood vessels and inhibits angiotensin II-mediated aldosterone production. It also reduces sodium and water retention and increases potassium excretion. All these actions collectively lower the blood pressure [7].

CHT (Fig. 1b) is 2-Chloro-5-[(1RS)-1-hydroxy-3-oxo-2,3-dihydro-1H-isoindol-1-yl]benzenesulfonamide [4]. CHT is a long-acting thiazide-like diuretic drug used for treating high blood pressure or edema caused by heart, kidney, or liver failure. It acts by inhibiting the sodium-chloride cotransporter through the renal tubular epithelium in the cortical diluting segment of the ascending loop of Henle [8]. AML (Fig. 1c) is a 1,4-dihydropyridine-3,5-dicarboxylate derivative [4]. AML inhibits the influx of calcium ions into vascular and cardiac muscles and is classified as a calcium channel blocker. AML is listed as an essential antihypertensive medicine by the World Health Organization (WHO). AML is widely regarded as a highly safe and exceptionally efficient therapy for hypertension and chronic stable angina [9].

Drug dissolution testing is a crucial analytical method for determining the quality of a product, analysing the behaviour of drug release, and identifying variations in the formulation and manufacturing process [10]. In addition, the dissolution testing holds significant importance in the regulatory assessment of drug product quality, particularly for solid oral dosage forms. Consequently, in-vitro dissolution monitoring emerges as a crucial indicator of the in-vivo performance of the drug. This testing procedure involves sampling at specific time intervals, which is subsequently followed by offline quantification of the released drug quantity [11]. Developing a dissolution methodology for pharmaceutical products with poor water solubility and drug combinations has proven to be difficult for the pharmaceutical industries [10]. The utilization of the high-performance liquid chromatography (HPLC) technology is a cornerstone in the processes involved in dissolution testing. The separation of HPLC provides time saving, high sensitivity, specificity, selectivity, and a broad linearity range. Due to the sensitivity of HPLC than the direct spectrophotometric approach, so the HPLC method is frequently utilised for drug product dissolution testing with extremely low potencies [12, 13].

To the best of our knowledge, only three reported methods were published for determination of the studied mixture including HPLC [14], high performance thin layer chromatography (HPTLC) [15], and UV spectrophotometry [16]. Also, few HPLC methods were published for cited drugs with other antihypertensive drugs [17, 18]. There is no single reported method applied for assay of the drug mixture in this commercial fixed dose combination (Telma-ACT^®^ Tablets) or for in-vitro dissolution studies. Hence, the objective of this study was to authenticate an HPLC–DAD method for quantifying TEL, CHT, and AML. Additionally, the developed method was utilized to assess the in-vitro release of these drugs from their fixed combined tablet dosage form (40/12.5/5 mg; TEL/CHT/AML). The proposed method can be applied for conducting dissolution studies on other formulations containing these compounds. Researchers within the analytical community strive to discover alternative options that can replace hazardous compounds with cleaner alternatives or, at the very least, minimize their quantity to a safe threshold [19, 20]. Various green metrics have been developed to assess and compare the environmental impact of analytical methods, acknowledging that the concept of "greenness" is relative rather than absolute. The level of environmental friendliness of the proposed method is evaluated through various metrics such as the green analytical procedure index (GAPI) [21–23], Analytical greenness metric for sample preparation (AGREEprep) [24], Analytical Eco-Scale (AES) [25], and the introduction of the analytical method greenness score (AMGS) [26]. Additionally, two newly developed assessments were applied including White Analytical Chemistry (WAC) which focuses on examining the attributes of whiteness, and the Blue Applicability Grade Index (BAGI) which examines the practically of the procedures [27–29].

## Experimental

### Apparatus and software

For chromatographic analysis; an Agilent 1260 Infinity II LC system, consisting of a quaternary pumping system (model G7111B), an auto-sampler (model G7129A), and photodiode array detector (model G7115A) from Agilent, Waldbronn, Germany, were utilized. Agilent openLab CDS Software was used for data acquisition and integration. A Jenway glass pH sensing electrode (Essex, UK) was operated for pH adjustments.

The dissolution testing was conducted utilizing the VanKel VK 7000 (USA) equipment equipped with the standard USP type-II paddle and six vessels.

AGREEprep open access software can be obtained from mostwiedzy.pl/AGREEprep. The AMGS calculator was obtained from https://www.acsgcipr.org/amgs, and blue assessment (BAGI) software from mostwiedzy.pl/bagi.

### Materials and reagents

Pure TEL and AML were helpfully provided by Global Napi Pharmaceuticals, Egypt with a certified purity of 99.58% and 98.75%, respectively. While, CHT was kindly supplied by EIPICO pharmaceuticals, Egypt with certified purity of 99.12%.

Commercially available Telma-ACT^®^ Tablets (40 mg/12.5 mg/5 mg), (BN: 30TTM008) were obtained from a local Indian pharmacy.

HPLC-grade of Acetonitrile and Methanol were supplied from Sigma-Aldrich (Germany). The Aquatron® water purification unit (A4000D, UK) was utilized to produce the in-house double distilled water. Analytical grade of orthophosphoric acid, potassium phosphate monobasic, and hydrochloric acid were purchased from Sigma Aldrich (Germany).

### Standard solutions

Three distinct stock solutions of TEL, CHT, and AML (500.0 µg/mL, each) were prepared by precisely weighing 50.0 mg of each pure analytical standard and transferring it into a 100-mL volumetric flask containing methanol as the solvent. The solutions were stirred for 10 min to ensure the complete dissolution of the standards. Subsequently, these solutions were safeguarded from light and stored at a temperature ranging from 2 to 8 °C.

### HPLC–DAD chromatographic conditions

Chromatographic separation was conducted using HPLC equipped with diode array detector (DAD). The separations were performed using an analytical column of Inertsil C_18_ (250 × 4.6 mm, 5.0 μm). The optimized mobile phase composed of a mixture of acetonitrile and 20.0 mM phosphate buffer (pH of 3.0 ± 0.1) in a ratio of 35:65, v/v. Prior to use, the mobile phase was filtered through 0.45-μm membrane filters, degassed, and then pumped at a flow rate of 1.0 mL/min in an isocratic manner. UV detection was carried out at 240.0 nm, and all measurements were performed at room temperature.

### Validation parameters

#### Construction of calibration curves and linearity range

The process of constructing calibration curves involved preparing six different standard solutions of TEL, CHT, and AML. These solutions were prepared by diluting the stock solutions of each compound with the mobile phase. The concentrations of the solutions ranged from 1.0–140.0 μg/mL for TEL, and 1.0–100.0 μg/mL for CHT and AML. After preparing the solutions, triplicate injections of 30 μL each were made, and the chromatographic conditions were followed. The peak areas obtained from the injections were then plotted against the corresponding concentrations of each component to assess linearity. Calibration curves were constructed using this data, and regression equations were calculated. The entire process was conducted in accordance with the ICH guidelines for assay validation [30].

#### Accuracy

The accuracy of the developed method was conducted by analysing five different concentration levels within the established linearity range for the drugs being studied, and each conducted three times. The accuracy was expressed by recovery percentages.

#### Precision

The precision of the developed method was assessed in terms of intra-day and inter-day precision. Intra-day precision for each drug was evaluated by calculating the relative standard deviation of three replicates at three different concentrations within the same day. Inter-day precision was assessed by calculating the relative standard deviation of three replicates at the same three concentrations on three consecutive days. The used concentrations were 5.0, 10.0, 40.0 μg/mL for TEL, 5.0, 12.5, 40 μg/mL for CHT, and 5.0, 20.0, 40.0 μg/mL for AML.

#### Specificity

The specificity of the proposed method was evaluated by analysis of several laboratory-prepared mixtures of the cited drugs in different ratios. Also, selectivity of the proposed method was demonstrated by the ability to quantify the cited drugs, simultaneously in Telma-ACT® Tablets without interferences from excipients.

#### Limit of detection (LOD) and limit of quantitation (LOQ)

LOD and LOQ of the proposed RP-HPLC method were calculated according to ICH guidelines using signal to noise ratio.

#### Robustness

The robustness of the HPLC–DAD method was evaluated through deliberate change in experimental conditions such as adjusting the buffer pH by ± 0.1 units, altering the mobile phase composition by ± 2%, or adjusting the flow rate by ± 0.1 mL/min. Robustness was assessed by calculating the %RSD of the response.

### Pharmaceutical formulation analysis

Twenty Telma-ACT^®^ Tablets (40 mg/12.5 mg/5 mg) were accurately weighed and their mean weight was estimated. Subsequently, the tablets were ground into a fine powder. The powdered tablets, equivalent to 40 mg of TEL, 12.5 mg of CHT, and 5 mg of AML, were then weighed and transferred into a 100-mL volumetric flask. The volume of the flask was adjusted to the mark using methanol. To ensure complete dissolution of the active ingredients, the contents of the flask were sonicated for a duration of 30 min. The resulting solution was then filtered through a Whatman filter paper. An aliquot of one mL from the filtered solution was transferred into a 10-mL volumetric flask. The volume of the flask was then completed with the mobile phase, resulting in a testing solution containing 40 μg/mL of TEL, 12.5 μg/mL of CHT, and 5 μg/mL of AML. This testing solution was subsequently subjected to assay analysis using the developed chromatographic method.

### In-vitro dissolution studies

Three dissolution media were prepared employing paddle (USP-II apparatus) at 75 rpm and bath temperature maintained at 37 ± 0.5 °C. The media were phosphate buffer solution (pH 7.5, 900 mL), water (900 mL), and 0.01 N HCl (500 mL). The dissolution samples were collected at 10, 15, 20, 30, 45, and 60 min. At each time interval, a 5-mL sample was withdrawn from each vessel using a syringe-filter (0.45 μm) and replaced with the same volume of fresh medium to keep a constant total volume. These aliquots were filtered and analysed using the developed HPLC–DAD method. The dissolution experiments were conducted in triplicate. The concentrations of the studied components in the test samples were determined by calculating the percentage of drug dissolved. This was achieved by using the respective calibration curve constructed in each medium.

## Results and discussion

### Optimization of chromatographic condition

To optimize the chromatographic conditions for the estimation of TEL, CHT, and AML in bulk, tablets and in vitro dissolution samples analysis, different conditions were performed. Three columns were tried for optimizing the separation including Intertsil ODS-3 (3.0 × 100 mm, 3.0 μm), Inertsil ODS-3 (250 × 4.6 mm, 5.0 μm), and Inertsil ODS-3 (4.6 × 150 mm, 5.0 μm). It was found that Inertsil C_18_ (250 × 4.6 mm, 5.0 μm) column achieved the best resolution for the separation of the studied drugs.

Taking in consideration the main objective of Green analytical chemistry to use green solvents that have low risk on the environment, different mobile phases with different ratios were tried. Initially, methanol was used as the organic phase in various ratios with phosphate buffer. However, this resulted in ineffective separation of the targeted drugs and the appearance of unsymmetrical and broad peaks. Using acetonitrile instead of methanol achieved better separation, thus may be due to its higher eluting power. Various buffer pH with different concentrations were examined, and 0.02 mM potassium dihydrogen phosphate buffer pH 3.0 ± 0.1 was the optimal with adequate separation of the components. The ratio selection of the acetonitrile and phosphate buffer solution in the mobile phase was challenging point, as by increasing the percentage of acetonitrile (> 35%) in the mobile phase, leading TEL, and CHT peaks to appear nearly to each other or superimposed and decrease their resolution. On the other hand, by decreasing percentage of acetonitrile in the mobile phase (< 35%), the retention time of the cited drugs increased and total run time increased. Consequently, the optimum ratio of acetonitrile and phosphate buffer was 35:65%v/v at a flow rate of 1.0 mL/min. Different wavelengths using the diode array detector were investigated according to the UV absorbance spectra of the studied drugs (Fig. S1). The studied wavelengths were 220.0, 230.0, and 240.0 nm. By comparing the three chromatograms for separation of the studied drugs, it was found that maximum sensitivity for TEL and CHT was at 220.0 nm, while for AML was 240.0 nm. UV detection at 240.0 nm was selected for quantification of all the cited compounds as it provided satisfactory results for all cited drugs and minimum noise for the analysis.

**Fig. 2:**
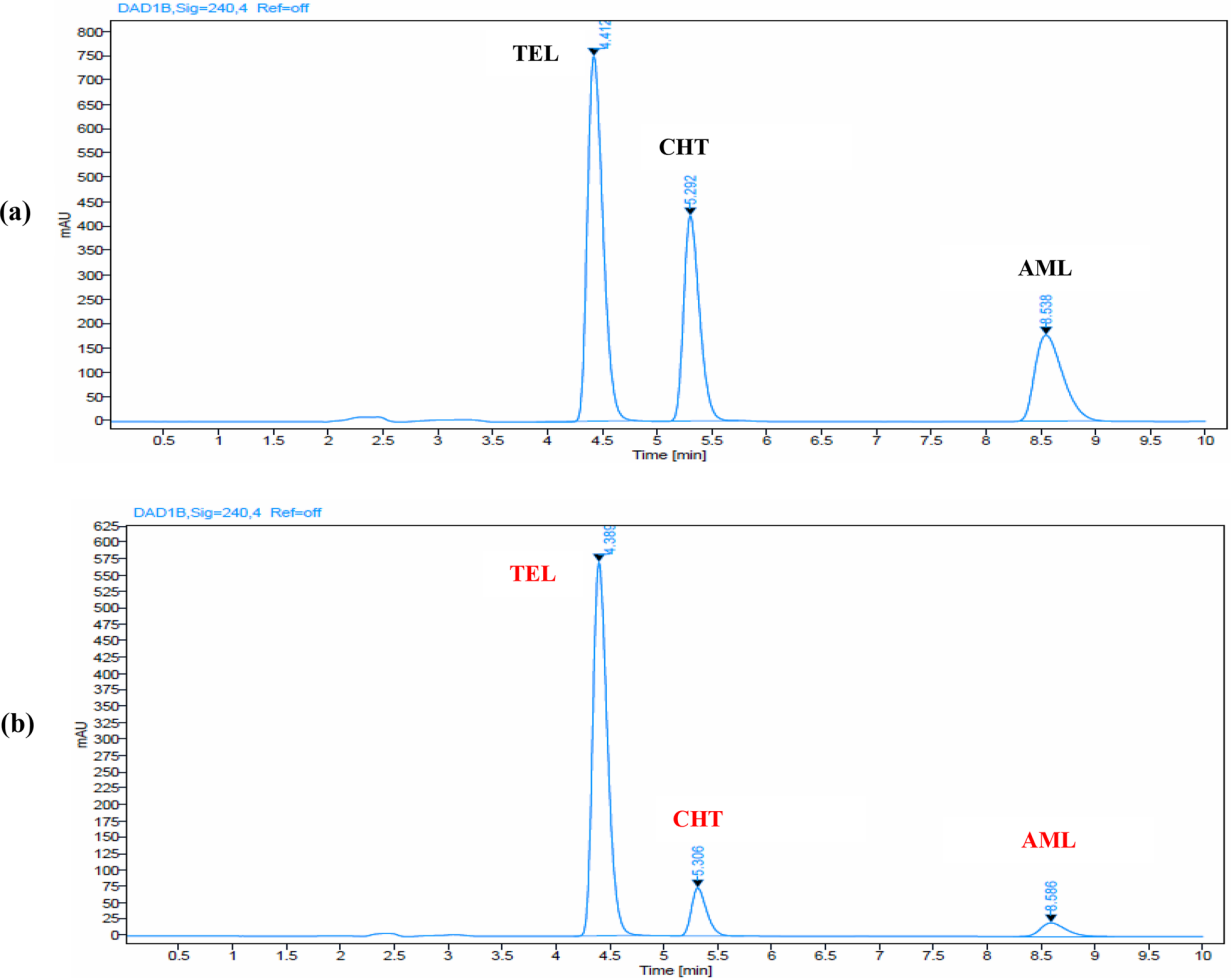
High performance liquid chromatograms of: **a** resolved prepared-lab mixture of 60.0 μg/mL telmisartan, 80.0 μg/mL chlorthalidone and 60.0 μg/mL amlodipine and **b** pharmaceutical dosage form (Telma-ACT® Tablets) containing 40.0 μg/mL telmisartan, 12.5 μg/mL chlorthalidone, and 5.0 μg/mL amlodipine

Different flow rates (0.8, 1.0, 1.2 mL/min) were used for the mobile phase. It was found that a flow rate of 1.0 mL/min resulted in satisfactory retention times and effective separation. Finally, upon using the optimum chromatographic conditions, sharp symmetric peaks with satisfactory baseline separation of TEL, CHT, and AML was achieved within 9 min, as shown in (Fig. 2a). The retention times of TEL, CHT, and AML were 4.3, 5.5 and 8.5 min, respectively.

### System suitability

Before each stage of validation, the chromatographic system's suitability was assessed according to Food and Drug Administration (FDA) guidelines [31]. Five injections of the standard preparation were measured, and the system's suitability was evaluated by calculating the capacity factor, tailing factor, height equivalent to a theoretical plate (HETP), and resolution, which confirmed its suitability as demonstrated in Table 1. All calculated parameters were found to be within the acceptable reference range.

**Table 1:** System suitability parameters of the proposed HPLC–DAD method

Parameter	TEL	CHT	AML	Reference value [31]
Retention time (min ± 0.2)	4.3	5.3	8.5	
Capacity Factor (k’)	1.05	1.52	3.05	
Selectivity (α)*	1.45 2.01			> 1
Resolution (R_s_)^*^	3.46 4.92			Rs > 2
Tailing factor (T)	1.15	1.21	1.24	T ≤ 2
Column efficiency (N)	5088	6875	6281	N ˃ 2000
Height equivalent to theoretical plate HETP (cm/plate)	0.0049	0.0036	0.0040	

*Selectivity and resolution factors are calculated between each two successive peaks

### Method validation

The validation of the proposed chromatographic methods has been conducted in compliance with the guidelines set forth by the International Council for Harmonisation (ICH). These parameters are shown in Table 2.

**Table 2:** Method validation parameters of the proposed HPLC–DAD method for determination of a ternary mixture of Telmisartan, Chlorthalidone and Amlodipine in pure form

Method parameter	TEL	CHT	AML
Range	1.0–140.0 μg/mL	1.0–100.0 μg/mL	1.0–100.0 μg/mL
Regression equations parameters			
Slope (b)^a^	120.2	52.27	52.73
Intercept (a)^a^	50.514	10.289	37.134
Correlation Coefficient (r)	0.9999	0.9999	0.9999
Accuracy^b^ (Mean ± SD)	100.52 ± 0.62	100.26 ​ ± 1.05	100.09 ± 1.17
Precision			
(%RSD)^c^	0.65	0.14	0.92
(%RSD)^d^	1.50	0.96	1.38
Specificity^e^	100.30 ± 0.98	99.87 ± 1.22	100.39 ± 1.11
LOD^f^	0.018	0.054	0.094
LOQ^f^	0.061	0.177	0.313
Robustness^g^	0.85	1.21	0.90

^a^Regression equation for HPLC: *A* = *a* + *bc*, where ‘A’ is the average peak area and ‘c’ is the concentration (μg/mL). Six calibration points, each conducted three times

^b^Mean recovery ± SD for five concentration values in between the calibration points in μg/mL, each conducted three times

^c^Intra-day precision [average of three different concentration of three replicates each (n = 9) within the same day], the concentrations were (5.0, 10.0, 40.0 μg/mL) for TEL, (5.0, 12.5, 40 μg/mL) for CHT, and (5.0, 20.0, 40.0 μg/mL) for AML

^d^Inter-day precision [average of three different concentration of three replicates each (n = 9) repeated on three successive days], the concentrations were the same as in intra-day precision

^e^ Mean recovery ± SD for determination of the drugs in laboratory prepared mixtures

^f^LOD and LOQ were calculated according to signal to noise ratio

^g^%RSD for the change in mobile phase ratio (± 2%), flow rate (± 0.1 mL/ min) and buffer pH (± 0.1)

The proposed HPLC–DAD method showed a linear relationship between the peak areas and the corresponding concentrations of TEL, CHT and AML. The calibration curves were constructed in the range of (1.0–100.0 μg/mL) for CHT and AML, while it was (1.0–140.0 μg/mL) for TEL. Adequate recoveries within acceptable percentage ranges were achieved by analysing five concentration levels within the established linearity range for the cited drugs, as presented in Table 2, and this confirms the high accuracy of the developed analytical method. Intraday precision and intermediate precision were evaluated by calculating RSD% of the peak responses and the results was within the accepted limit. Method specificity was verified by comparing chromatograms of blank, standard, and sample solutions (Telma-ACT^®^ Tablets). The resulted chromatograms showed no chromatographic interference peaks from the excipients containing TEL, CHT, and AML as shown in Fig. 2b. Additionally, the proposed method was found to be specific through accepted recovery percentage from multiple laboratory prepared mixtures with various drug concentrations as shown in Table S1. No additional peaks were observed beyond those corresponding to TEL, CHT, and AML, confirming the method's specificity for their assay. Moreover, the specificity of a chromatographic method as a separation technique can be demonstrated by utilizing the spectral display and peak purity monitor using online recording with a diode array detector.

The estimation of LOD and LOQ were calculated by signal to noise ratio method. The values of LOQ and LOD of the studied drugs are displayed in Table 2. For evaluation of robustness of HPLC–DAD method, experimental conditions were slightly and deliberately changed such as small change in buffer pH ± 0.1, the mobile phase composition ± 2%, or flow rate (± 0.1 mL/min). Robustness was evaluated by calculating %RSD of response and the obtained results were ensuring sufficient robustness of the proposed method.

### Application of the proposed method on pharmaceutical formulation

The validity and applicability of the proposed HPLC–DAD method for the quantitative analysis of TEL, CHT, and AML in Telma-ACT® Tablets, without interference from excipients, has been confirmed, Fig. 2b. The obtained results align well with their expected content, as evidenced in Table 3, thereby affirming the suitability of the suggested HPLC technique for the regular determination of the analytes in their combined dosage form. Furthermore, the standard addition procedure was implemented to assess the validity of the developed method, which revealed no interference from excipients. The results obtained are presented in Table 3.

**Table 3:** Results obtained by applying the proposed HPLC–DAD method for the determination of Telmisartan, Chlorthalidone and Amlodipine in Telma-ACT^®^ Tablets and application of standard addition technique

Pharmaceutical formulation	HPLC–DAD method				
	Drug	%Found ± SD^a^	Standard addition technique		
			Claimed (μg/mL)	Pure added (μg/mL)	%Recovery of the pure added amount^b^
Telma-ACT^®^ Tablets (Each tablet was Labelled to Contain 40 mg TEL, 12.5 mg CHT & 5 mg AML) (BN: 30TTM008)	TEL	101.46 ± 1.30	40	20.0	99.05
				40.0	101.75
				80.0	100.71
			Mean ± SD		100.50 ± 1.36
	CHT	98.77 ** ± **0.77	12.5	6.25	98.40
				12.5	99.20
				25.0	100.12
			Mean ± SD		99.24 ± 0.86
	AML	101.35 ± 1.33	5	2.5	100.40
				5.0	98.00
				10.0	99.80
			Mean ± SD		99.40 ± 1.25

^a^Average of five determinations

^b^Average of three experiments

Statistical comparison between the proposed HPLC method, and the reported method [16], revealed no significant difference as pointed in Table S2 which indicates that the calculated t-test and F-value are less than the tabulated ones.

### In-vitro dissolution studies

Since, there is no definite dissolution medium for the dissolution test of this co-formulated dosage form, dissolution tests for Telma-ACT^®^ Tablets were performed to investigate the release of the TEL, CHT, and AML drugs in three different media according to USP monograph of each drug separately. The recommended USP dissolution medium is phosphate buffer pH 7.5 for TEL, 0.01N HCl for AML and water for CHT [5].

**Fig. 3:**
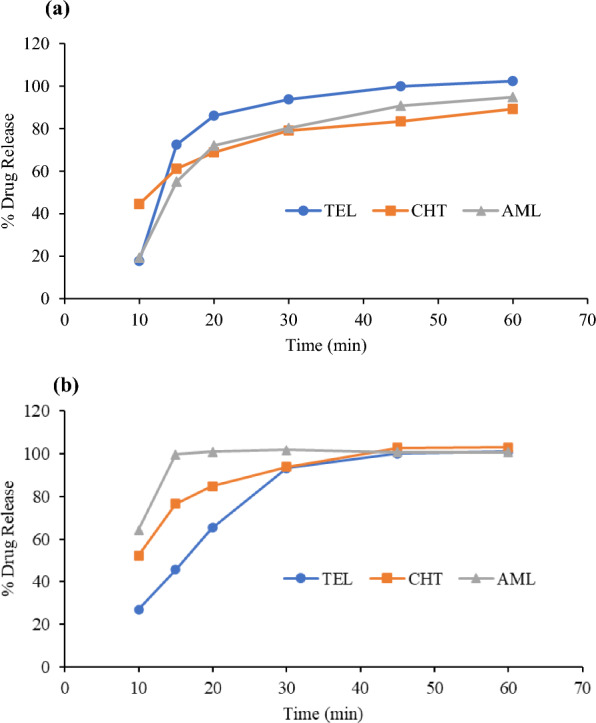
In-vitro dissolution profile of TEL, CHT, and AML in Telma-ACT^®^ Tablets in **a** phosphate buffer pH 7.5 and **b** 0.01N HCl

It was found that more than 85% of TEL was released from tablets in 0.01N HCl and in phosphate buffer within 30 min, with complete release (about 100%) achieved at 45 min. For CHT, 85% release was achieved in 0.01N HCl at 20 min, with complete release (102%) at 45 min. However, the release of CHT in phosphate buffer was about 84% at 45 min and about 89.2% at 60 min. For AML, complete and rapid release (99.7%) was achieved within 15 min in 0.01N HCl. In phosphate buffer, the release of AML was 80% at 30 min and 95% at 60 min. Practical results revealed that the average percentage of drug release during the in-vitro dissolution of combined tablets was imprecise and inaccurate when using water as the dissolution medium. This could be attributed to the inadequate water solubility of TEL and AML. In contrast, the average percentage of drug release in Telma-ACT^®^ Tablets was acceptable and precise in phosphate buffer pH 7.5 and 0.01N HCl, as shown in Figs. 3a and b.

Consequently, the 0.01N HCl media was the medium of choice for dissolution testing of TEL, CHT and AML drugs in tablets. The dissolution medium's pH level falls within the pH range of the gastrointestinal tract, making it a bio-relevant environment that mimics the conditions of the gastrointestinal tract. The established HPLC–DAD method was efficiently applied for determination of average percentage drugs released within 30 min for in-vitro dissolution of tablets containing combination drug product. The in-vitro dissolution studies revealed that more than 90% of labelled amounts of TEL, CHT and AML were released within 30 min from their fixed combination tablet dosage form.

### Greenness, whiteness, and blueness assessment tools

#### Analytical Eco-Scale (AES)

The Eco-Scale tool, which utilizes a mathematical formula, is employed to assess the score of a procedure based on penalty points. Through this calculation, a value is generated that represents the level of environmental friendliness of the process by subtracting the total penalty points from 100. These scores are determined on an environmental sustainability scale, where a score of 75 or above indicates "excellent" performance, a score of 50 or higher signifies "acceptable," and a score of 50 or lower denotes "in adequate." Table 4 displays the penalty points and the results of the AES computations for the proposed technique. As per the AES evaluation, the approach achieved a score of 75, indicating that it is an outstanding environmentally friendly method.

**Table 4:** Assessment of the proposed chromatographic method greenness according to Analytical Eco-Scale, GAPI and AGREEprep, blueness via BAGI tool and whiteness assessment by RGB 12 model

Eco-Scale assessment		GAPI Assessment^b^	AGREEprep^c^	BAGI Assessment^d^	RGB12 algorithm^e^
Reagents	Penalty points (PPs)				
Acetonitrile	4				
Potassium dihydrogen phosphate	4				
Hydrochloric acid	4				
Methanol	6				
Instrument					
Energy consumption^a^	1				
Occupational Hazard	0				
Waste	6				
Total PPs	25				
Analytical Eco-Scale Total Score	75				
Comment	Excellent green analysis				

^a^Calculated as: run time × flow rate for HPLC method

^b^GAPI Assessment evaluated according to Green Analytical Procedure Index parameters description [21]

^c^AGREEprep Assessment evaluated by using Analytical GREEnness Metric approach and software [24]

^d^Blue Applicability Grade Index (BAGI) as a new metric tool for evaluating the practicality of an analytical method [29]

^e^RGB12 algorithm for whiteness evaluation [27]

#### Analytical Greenness metric for Sample Preparation (AGREEprep)

AGREEprep, a recently endorsed assessment tool, represents the inaugural measure explicitly designed for evaluating sample preparation processes. It operates across ten consecutive assessment periods, aligning with the ten principles of the Green Sample Preparation (GSP) [24]. Distinguishing itself from previously published metrics, AGREEprep sets itself apart focusing specifically on the sample preparation phase, providing precise and targeted assessments of the environmental impact associated with these procedures. Furthermore, AGREEprep evaluation extends beyond assessing environmental friendliness alone; it also helps identify methodological strengths and weaknesses, thereby promoting the adoption of more environmentally sustainable sample preparation techniques.

The AGREEprep open access software and source code can be found at mostwiedzy.pl/AGREEprep, and git.pg.edu.pl/p174235/agreeprep, respectively. AGREEprep software calculates scores for each of the ten evaluation periods, ranging from 0 to 1. The highest and lowest scores represent the best and worst achievements. These scores are then weighted and combined to generate a combined score; similarly, ranging from 0 to 1, where 1 indicates the best performance. Once the evaluation is complete, the AGREEprep software makes a circular icon. This icon consists of a central circle indicating the ten bars with total score, each need entering data for a specific step of the assessment. After the assessment, each component changes its color, leading it easier to identify the strengths and weaknesses of the process and their impact on the final result. According to Table 4, the AGREEprep score for the proposed method is 0.64, indicating its environmentally friendly and sustainable nature.

#### Green analytical procedure index (GAPI)

The Green Analytical Procedure Index (GAPI) [21] is a newly developed tool that incorporates features from Analytical Eco-Scale to give qualitative and comprehensive information. GAPI evaluates the ecological footprint of an entire methodology, covering every stage from sample collection to the final determination. Employing a distinctive symbol comprised of five pentagrams, this tool systematically assesses and quantifies the environmental impact linked to each step in the methodology. GAPI utilizes a tri-color system, with red denoting high environmental risk, while yellow and green signify lower risk and enhanced environmental sustainability, respectively. By providing comprehensive insights into analyzed methods, GAPI ensures that users and readers gain an easily comprehensible perspective. In Table 4, the GAPI index ranks the suggested approach for examining the drugs under consideration. The GAPI index for the suggested environmentally friendly method revealed green and yellow pentagrams, with only one red pentagram. This indicates that the proposed approach is environmentally advantageous, due to reduced waste generation, as mentioned in Table S3.

#### Analytical method greenness score (AMGS)

AMGS is considered one of the newest comprehensive analytical techniques used for green assessment. The AMGS offers a convenient and user-friendly solution that checks the inputs from three key domains: environmental safety, waste, and energy consumption [26]. The website (https://www.acsgcipr.org/) openly shares the guidelines and provides access to the tool for widespread utilization. To effectively utilize the AMGS calculator, one must consider the current environment, health, and safety index scores. These scores are used to determine the safety index of the solvent, the solvent health index, and the environmental index score for the solvent. Additionally, the calculator provides a preset drop-down menu for selecting common solvents as mobile phases or solvents for regular dissolution. The necessity of manually entering data, including elution time, flow rate, and gradient parameters, is essential. The AMGS tool also offers a range of instrumental types to choose from through its slide-down option. The solvent usage section of the AMGS tool provides essential information on the methodology, sample preparation, system suitability, sensitivity, selection of mobile phase, and instrumental conditions. Utilizing environmentally friendly solvents and more user-friendly instruments leads to a more sustainable and reduced AMGS score. The calculator will indicate the three different sections of AMGS through a colour shift from yellow, orange to red if they constitute 50% or more of the overall AMGS input. The assessment of the analytical method being considered produces a score that reveals the impact of each parameter on the calculator score, along with color-coding. The color-coding indicates the primary parameter influencing the score. Upon inputting the necessary data into the AMGS tool for evaluating greenness, a greenness score of 290 was acquired. The solvent energy score and solvent EHS score demonstrated greenness, while the instrument energy score indicated orange, suggesting that further reduction in instrument energy consumption is required in accordance with GAC principles (Fig. S2). However, the overall method attained a relatively lower greenness score, signifying that the method is indeed environmentally friendly (as the greenness score decreases, the level of greenness increases).

#### Whiteness assessment

The White Analytical Chemistry (WAC) serves as a comprehensive and thorough tool for assessing various analytical methods. WAC composes of three crucial sustainable criteria: method effectiveness, environmental impact, and economic considerations. The accompanying Excel worksheet simplifies the process of achieving the desired outcome.

The concept of attaining a "white" outcome in sustainability analysis is derived from the combination of three primary colors: red, green, and blue. Red signifies the effectiveness of the analytical approach, covering aspects such as the scope of application, detection and quantification limits, accuracy, and precision. Green corresponds to the GAC 12 principles, addressing reagent toxicity, quantity of reagents used, waste management, energy consumption, and direct impacts. These factors are pivotal considerations. Blue involves inputting information related to cost and time efficiency, requirements, and operational simplicity. To evaluate the sustainability of two analytical methods using the WAC metric, a table with three columns (red, green, blue) is provided in an accessible Excel worksheet. Based on the given data, results are calculated as a numerical value out of 100, indicating the sustainability level of the analytical method. The whiteness diagram illustrates the proportions of specified colors and their combination leading to the formation of the color white. Analyzing the chart allows us to assess the efficiency and sustainability of the applied analytical approach. An optimal sustainable technique should exhibit elevated percentages for all three colors, with a particular focus on attaining a substantial proportion of white. The obtained results for whiteness profile of the proposed method were shown in Table 4. In reference to the red color category, the suggested method achieved a a notable score of 97.5%, owing to its impressive sensitivity demonstrated by the limits of detection and quantification. Concerning the green color category, the proposed method achieved a score of 82.9% by efficiently reducing solvent usage, energy consumption, and waste generation. Additionally, with respect to the blue category, the proposed method secured a score of 84.6%. Combining these individual color scores, the proposed method attained an overall whiteness score of 88.3%, clearly indicating its enhanced sustainability.

#### Blueness assessment

The Blue Applicability Grade Index (BAGI) [29] is introduced as a novel metric for assessing the usability of an analytical technique in practical settings. BAGI serves as a complementary measure to the established green metrics, with a primary focus on the pragmatic aspects within White Analytical Chemistry. This index evaluates ten key attributes, encompassing the nature of analysis, simultaneous determination of analytes, sample throughput per hour, reagent and material specifications, instrumentation needs, concurrent sample treatment capacity, pre-concentration prerequisites, level of automation, sample preparation methodology, and sample quantity requirements.

By assessing these attributes, a pictorial representation in the form of an asteroid diagram is generated, along with the corresponding score. To enhance the accessibility of the metric, a user-friendly, open-source application has been developed, accessible at mostwiedzy.pl/bagi. Additionally, a web-based application is available at bagi-index.anvil.app for easier utilization.

The comprehensive assessment result is visualized through an asteroid-like pictogram featuring a central numerical value. The color spectrum of this pictogram indicates the degree of alignment of the method with predefined criteria. Specifically, dark blue represents high alignment, blue indicates moderate alignment, light blue suggests low alignment, and white signifies no alignment. The numeric value within the central portion of the BAGI pictogram represents the overall score assigned to the analytical method, ranging from 25 to 100. A score of 25 indicates poor applicability, while a score of 100 denotes exceptional method performance. For an analytical method to be deemed "practical," it must achieve a minimum score of 60, a threshold widely endorsed within the framework of this assessment tool.

The evaluation of the proposed HPLC method was carried out using the BAGI tools, resulting in a favourable score of 80, signifying its strong applicability as shown in Table 4. This high score was due to using the common available reagents and simple instrument available in each research lab. The sample preparation was minimal, involving tasks like dilution and filtration, with less than 30 μL of sample needed for analysis. The total run time was less than 10 min, yielding analysis of six samples per hour. Remarkably, pre-concentration was unnecessary, and the required sensitivity was achieved directly. The BAGI assessment confirms that the proposed method offers significant benefits in terms of time and cost efficiency, hazard reduction, and overall effectiveness.

## Conclusion

In recent years, the scope of pharmaceutical dissolution testing has significantly expanded, extending beyond the quality control of dosage forms to play a crucial role in bioavailability testing and the screening of various formulations. Dissolution testing plays a crucial role in the advancement of orally administered solid dosage forms. In the present day, dissolution testing stands as a vital tool in the formulation development of novel chemical entities. A new, environmentally friendly, cost-effective, and selective HPLC–DAD method has been developed and validated for the separation and quantification of TEL, CHT, and AML. Adhering to ICH guidelines, the proposed method has demonstrated accuracy, specificity, robustness, and sensitivity. This method has been successfully applied to measure the in-vitro release of the studied drugs from their fixed combined tablet dosage form (40/12.5/5 mg; TEL/CHT/AML). The proposed study presents the greenness profile using four evaluation metrics, namely GAPI, AGREEprep, AES, and AMGS calculator, whiteness assessment via WAC metrics, and blueness assessment via BAGI tool. The aim of evaluating these tools is to assess the effectiveness in interpreting the results and providing both optical and numerical outcomes. The purpose of this assay is to provide data to pharmaceutical researchers involved in developing novel bio-relevant dissolution media and predicting the in vivo effectiveness of drugs with low solubility.

## Supplementary Information

Supplementary Material 1.

## Data Availability

All data generated or analysed during this study are included in this published article.

## Abbreviations

AES: Analytical Eco-Scale
AGREEprep: Analytical greenness metric for sample preparation
AMGS: Analytical method greenness score
AML: Amlodipine besylate
BAGI: Blue Applicability Grade Index
BP: British Pharmacopoeia
CHT: Chlorthalidone
DAD: Diode array detector
FDA: Food and Drug Administration
GAPI: Green Analytical Procedure Index
GSP: Green Sample Preparation
HETP: Height equivalent to a theoretical plate
HPLC: High performance liquid chromatography
HPTLC: High performance thin layer chromatography
ICH: International Council for Harmonisation
LOD: Limit of detection
LOQ: Limit of quantification
TEL: Telmisartan
USP: United States Pharmacopeia
WAC: White Analytical Chemistry
WHO: World Health Organization
