# Supplementary material for: A sustainable HPLC method coupled with diode array detection for versatile quantification of telmisartan, chlorthalidone and amlodipine in a fixed-dose antihypertensive formulation and dissolution studies
Source: BMC Chem. 2024 Sep 12;18(1):166. doi: 10.1186/s13065-024-01276-2 (PMC11391801; doi:10.1186/s13065-024-01276-2)
Supplement: Supplementary file 1 — Supplementary Material 1. [file 13065_2024_1276_MOESM1_ESM.docx]

**Table S1:** Results obtained by applying the proposed HPLC-DAD method for the determination of telmisartan, chlorthalidone and amlodipine in laboratory-prepared mixtures.

| AML | | | CHT | | TEL | | | Drug |
| --- | --- | --- | --- | --- | --- | --- | --- | --- |
| **Recovery%** | **Taken**  **(μg/mL)** | **Recovery%** | | **Taken**  **(μg/mL)** | | **Recovery%** | **Taken**  **(μg/mL)** | **Sample no.** |
| 101.22 | 5.00 | 98.98 | | 5.00 | | 100.75 | 5.00 | **1** |
| 100.68 | 10.00 | 98.59 | | 20.00 | | 98.83 | 80.00 | **2** |
| 99.31 | 60.00 | 100.64 | | 80.00 | | 101.34 | 60.00 | **3** |
| 99.15 | 50.00 | 101.56 | | 30.00 | | 99.84 | 120.00 | **4** |
| 101.58 | 5.00 | 99.59 | | 12.50 | | 100.72 | 40.00 | **5*** |
| **100.39 ± 1.11** |  | **99.87 ± 1.22** | |  | | **100.30 ± 0.98** |  | **Mean**  **± SD** |

*^Mixture 5 covers the dosage form ratio TEL: CHT: AML (40:12.5:5).^

**Table S2.** Statistical analysis of the results obtained by the proposed HPLC-DAD method and the reported method for the determination of Telmisartan, Chlorthalidone and Amlodipine in their pure forms [16]**.**

| Parameters | HPLC-DAD method | | | Reported method [16] | | |
| --- | --- | --- | --- | --- | --- | --- |
|  | **TEL​** | **CHT** | **AML** | **TEL​** | **CHT** | **AML** |
| Mean | 100.49 | 99.75 | 100.42 | 99.99​ | 99.99 | 100.15 |
| SD | 1.38​ | 1.20 | 1.17 | 1.19 | 0.71 | 1.32 |
| n | 6 | 6 | 6 | 6 | 6 | 6 |
| Variance | 1.90 | 1.44 | 1.38 | 1.42 | 0.50 | 1.74 |
| Student’s t-test (2.228)^*^ | 0.574 | 0.497 | 0.416 | ------- | ------- | ------- |
| F value (5.05)^*^ | 1.34 | 2.88 | 1.26 | ------- | ------- | ------- |

^*^ The values in the parenthesis are the corresponding theoretical values of t and F at P = 0.05.

**Table S3:** GAPI evaluation pictogram for the proposed method.

| **Category** | **Proposed method** | |
| --- | --- | --- |
| Circle in the middle of GAPI: procedure for qualification and quantification | 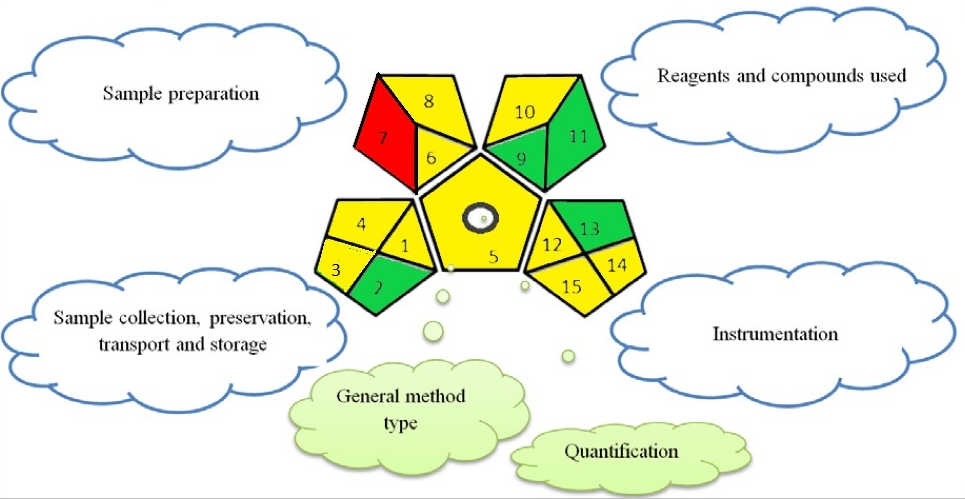 | |
| **Sample Preparation** | | |
| Collection (1) | On-line ISE (Yellow) | |
| Preservation (2) | No Need for preservation(Green) |  |
| Transport (3) | Require (yellow) |  |
| Storage (4) | Storage under normal condition (Yellow) | |
| Type of method [direct or indirect] (5) | Simple procedure as filtration (Yellow) | |
| Scale of extraction (6) | Micro-extraction (Yellow) | |
| Solvents/reagents used (7) | Non-green solvents/reagents used (Red) | |
| Additional treatments (8) | Simple treatments (clean up, solvent removals) (Yellow) | |
| **Reagents and solvents** | | |
| Amount (9) | Less than 10 mL (Green) | |
| Health hazard (10) | Moderately toxic (Yellow) | |
| Safety hazard (11) | No special hazards (Green) | |
| **Instrumentation** | | |
| Energy (12) | Less than 1.5 kWh per sample (Green) | |
| Occupational hazard (13) | Hermetic sealing of analytical process (Green) | |
| Waste (14) | Less than 10 mL (Yellow) | |
| Waste treatment (15) | No recycling (Yellow) | |

**AML**

**CHT**

**TEL**

**Figure S1:** Overlaid spectra of TEL (―), CHT, (-----) and AML (-----) (10.0 µg/mL, each).


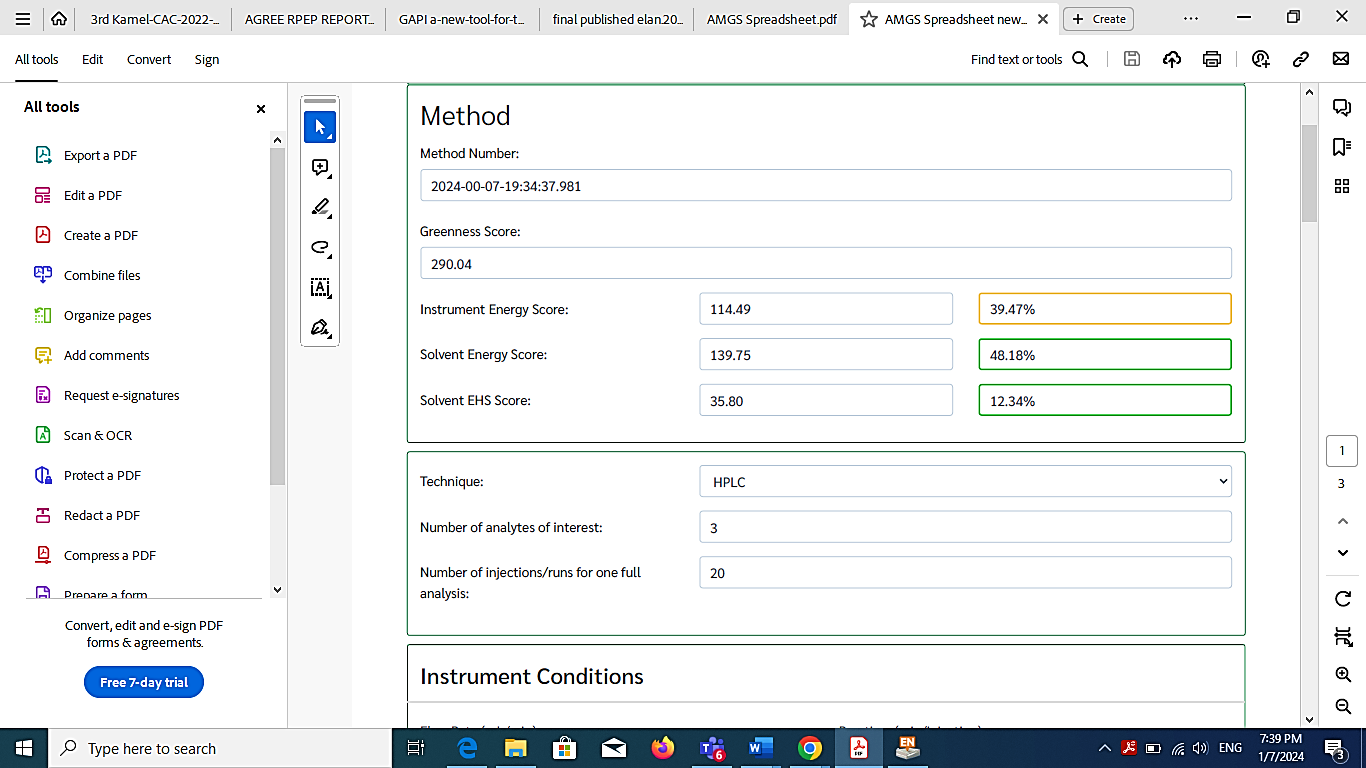


**Figure S2:** The greenness profile of proposed method using AMGS Calculator.
